# Effects of Vortioxetine on Sleep Architecture of Adolescents with Major Depressive Disorder

**DOI:** 10.3390/clockssleep5040042

**Published:** 2023-10-24

**Authors:** Zuzana Mlyncekova, Peter Hutka, Zuzana Visnovcova, Nikola Ferencova, Veronika Kovacova, Andrea Macejova, Ingrid Tonhajzerova, Igor Ondrejka

**Affiliations:** 1 Clinic of Psychiatry, Jessenius Faculty of Medicine in Martin, University Hospital Martin, Comenius University in Bratislava, Kollarova 2, 03601 Martin, Slovakia; mlyncekova3@uniba.sk (Z.M.); hutka6@uniba.sk (P.H.); kovacova400@uniba.sk (V.K.); macejova5@uniba.sk (A.M.); 2 Biomedical Centre Martin, Jessenius Faculty of Medicine in Martin, Comenius University in Bratislava, Mala Hora 4D, 03601 Martin, Slovakia; zuzana.visnovcova@uniba.sk (Z.V.); nikola.ferencova@uniba.sk (N.F.); 3 Department of Physiology, Jessenius Faculty of Medicine in Martin, Comenius University in Bratislava, Mala Hora 4C, 03601 Martin, Slovakia; ingrid.tonhajzerova@uniba.sk

**Keywords:** vortioxetine, REM suppression, depression, adolescent, sleep architecture, polysomnography

## Abstract

The relationship between depression and insomnia is bidirectional and both conditions need to be treated adequately, especially in a vulnerable neurodevelopmental stage of adolescence. This study aimed to evaluate the effects of antidepressant treatment using vortioxetine (VOR) on the sleep architecture of depressed adolescents by using video-polysomnography (v-PSG), which has not been researched before. The v-PSG was performed on 30 adolescent in-patients (mean age of 15.0 years ± 1.5 SD, 21 girls) treated with VOR (dosage of 10/15/20 mg/day) administered orally once a day, before and after VOR treatment. The evaluated parameters were conventional sleep parameters, sleep fragmentation parameters, and selected spectral power indices. Symptoms of depression and insomnia before and after the treatment period were evaluated using valid and reliable questionnaires (the Children´s Depression Inventory and the Athens Insomnia Scale). Depressed adolescents showed higher REM latency and decreased REM sleep percentage after treatment than before the treatment period (*p* = 0.005, *p* = 0.009, respectively). Our study revealed REM suppression (increased REM latency and reduced REM sleep percentage), indicating altered sleep architecture as a potential result of VOR treatment, which seems to be dose-dependent.

## 1. Introduction

### 1.1. Depression and Insomnia in Adolescents

Major depressive disorder (MDD) and co-occurring insomnia represent a serious concern, particularly in the adolescent period. The prevalence of depression in children and adolescents is estimated to be 14.3% [1] and the prevalence of insomnia is estimated to be 51–74% in clinically depressed adolescents [2]. In this aspect, recent scientific research is focused on their bidirectional relationship [2,3]. More specifically, sleep disturbances in adolescents have been associated with a more severe clinical course of depression (i.e., suicidal behavior, worse psychosocial functioning, and risk of recurrence) [2]. Due to the rising incidence of both disorders, it is crucial to understand the underlying pathophysiology (e.g., disrupted neuroplasticity and neurotransmitter dysbalance), especially in the vulnerable adolescent age period characterized by developmental changes and sensitivity to endogenous and exogenous factors [3,4]. Sleep architecture reflects not only physiological neurodevelopmental changes but also the effect of antidepressant treatment. The major changes in sleep during adolescence manifest as a decrease in total sleep time, a decrease of the N3 stage (represented by slow wave sleep, SWS), an increase in daytime sleepiness, and a circadian shift towards later hours [2]. With respect to depression, there are several consistent sleep alternations observed in depressed adolescents but expressed to a lesser degree when compared to sleep studies on adults. It is predominantly rapid eye movement (REM) sleep disinhibition, which can be influenced by antidepressants, as discussed in the following paragraphs.

### 1.2. Sleep Abnormalities in Adolescent Depression

There are several sleep electroencephalogram (EEG) biomarkers registrable by a polysomnographic study that correlate with a predisposition to developing the affective disorder, with the severity of depression, and have predictive value of response to treatment [2,5,6]. The most prominent ones are REM sleep abnormalities, reduction of SWS, and impaired sleep continuity (presented by increased sleep latency, sleep fragmentation by increased arousals, and wakefulness after sleep onset) [7]. More specifically, the REM disinhibition and its instability characterized by short REM latency (REM-L), increased REM sleep duration (REM%), increased REM density (RD), and REM fragmentation (the index of the total number of arousals during REM sleep/REM sleep duration in hours) is considered not only epiphenomena but to be a crucial biomarker of endogenous depression [2,6,8]. Moreover, restless REM sleep is deeply involved in the alternation of synaptic plasticity in limbic circuits leading to emotional distress and subsequent vulnerability to developing mood disorder [9]. Similarly, based on the knowledge that REM sleep has an important role in memory consolidation, emotional memory processing, stress response, reward, and energy homeostasis, chronic REM sleep alternations contribute to the pathophysiology and manifestation of mood disorders [10]. Thus, REM sleep disturbances represent a relevant clinical hallmark of depression and can be modified by pharmacologic therapy [6]. Moreover, periodic limb movements during sleep (PLMS) characterized as involuntary repetitive and stereotypical limb movements [11] was reported to be associated with psychiatric disorders including depression [12] already at adolescent age [13].

### 1.3. The Effects of Antidepressants on Sleep Architecture

The principal therapeutic intervention to manage symptoms linked to depression and insomnia is psychopharmacotherapy. However, antidepressants (ADs) are reported to have distinct effects on sleep architecture [7,14,15]. Some can eventually lead to sleep disturbances and contribute to a vicious circle. In general, the enhanced serotoninergic tone caused by ADs via interaction with the serotonin (5-HT) receptors potentiates cortical activity, and arousal system during sleep and suppresses REM sleep. This effect on REM sleep is typical and consistent for selective serotonin reuptake inhibitors (SSRIs) (except for escitalopram), serotonin-norepinephrine reuptake inhibitors (SNRIs), and tricyclic antidepressants (except for trimipramine) [6,7]. The resulting subjective sleep effect is variable since ADs (mostly SSRIs, the first-line treatment of depression in adolescence) might induce other sleep disturbances such as periodic limb movements, restless leg syndrome, or daytime somnolence [14]. Another hypothesis is that the REM-suppressing effect is distinct and not immediately connected to the antidepressant effect, as seen in acute administration of ADs, and may be related to dysfunction of brain structures (such as the limbic system) that are involved in both the REM sleep and mood regulation [6,7]. Furthermore, REM suppression was suggested to predict the treatment response [6]. In contrast, the effects of ADs on non-rapid eye movement (NREM) sleep and sleep continuity differ by interaction with specific receptors, as described in our previous review article by Hutka et al. [15]. Additionally, antidepressant treatment can trigger PLMS [16]; however, there is virtually no information on AD-induced PLMS at the adolescent age. Thus, this led to our interest in researching a novel AD such as VOR in association with sleep.

### 1.4. Vortioxetine—Pharmacokinetic/Molecular Mechanisms Related to Sleep

VOR, acting as multimodal serotoninergic AD, blocks serotonin transporter (SERT), 5-HT_3_, 5-HT_7_ and 5-HT_1D_ receptor antagonist, 5-HT_1B_ receptor partial agonist, and 5-HT_1A_ receptor agonist, and as a result, increases levels of dopamine, acetylcholine and noradrenaline in the frontal cortex and ventral hippocampus [17]. According to several clinical studies, VOR is characterized by antidepressant, anxiolytic, and procognitive effects due to changes in synaptic neuroplasticity, in an effective dose ranging from 5 to 20 mg (i.e., from 50 to 80% SERT occupancy) [18]. VOR shows linear and time-independent pharmacokinetics, with a mean T_max_ of 7–8 h and a mean elimination half-life of 57 h upon oral administration [19,20], which reduces the risk of discontinuation syndrome. VOR has a moderate oral bioavailability of 75% and an extensive tissue distribution. VOR is primarily metabolized by cytochrome P450 enzymes, mostly to an inactive major metabolite, with no clinical relevance with respect to sex, age, race/ethnicity, body size, or hepatic or renal function [20]. Notably, the treatment of depression by VOR in the pediatric population is off-label, but a long-term, open-label, clinical trial [21] reported its safety and efficacy in children and adolescents. However, the effectiveness of VOR remains controversial, since another study by the same author found no significant difference between VOR and placebo [22]. In general, VOR has proved its efficacy and tolerability in different studies [23]. Since cognitive impairment is an important symptom of MDD and is linked to sleep quality, it merits more attention in the context of VOR treatment. Moreover, it is suggested that, mostly through antagonism on the 5-HT_3_ receptor, it has a significant procognitive effect via modulation of neurotransmission in the medial prefrontal cortex and via enhancement of neurogenesis and neurotrophic processes in the hippocampus, as found in animal models [24]. In the quantitative EEG analysis, VOR increases low and high frontal cortical frequency ranges, which is also associated with cognitive processing enhancement [25,26]. According to functional imaging measurements, VOR shows direct positive effects on neural activity in typically overactive regions during acute episodes of MDD (such as dorsolateral prefrontal cortex and hippocampus) related to the decrease of subjective cognitive functions [23]. Apart from its procognitive effect, VOR has been intensively studied for its potential analgesic and anti-inflammatory effects [23]. Focusing on its sleep effect, there are only a few studies that assess the clinical effects of VOR on subjective sleep quality in positive correlation with depressive symptoms [27,28]. Only abnormal dreams, but not treatment-emergent insomnia or somnolence have been previously associated with VOR treatment in clinical trials [29]. To our knowledge, there are only two polysomnographic studies (one experimental in rats and one in healthy humans) that demonstrated REM-suppressing effects of VOR [17,30]. By increasing the levels of serotonin, one of the key regulators of the sleep–wake cycle, paradoxically VOR seems to be less fragmenting sleep compared to activating SSRIs or SNRIs and has a less intense REM-suppressing effect, indicating its different pharmacological profile [17]. Although this effect is particularly attributed to 5-HT3 receptor antagonism [18,30], other mechanisms are also suggested (discussed below). However, the current knowledge of VOR´s effects on sleep is limited (see Table 1).

Based on these studies, we hypothesized REM-suppressing effects of VOR in adolescents with major depression. Thus, we aimed to evaluate the effects of VOR on sleep architecture, especially its effects on REM sleep, by means of a polysomnographic study. To the best of our knowledge, this is the first study to evaluate VOR´s effects on sleep structure in adolescent patients with major depression and can contribute to new findings essential for precise pharmacotherapy.

## 2. Results

### 2.1. Comparison of Questionnaire Indices before and after Treatment

Both evaluated questionnaire indices—AIS, and CDI—were significantly lower after treatment (v-PSG II) compared to before the treatment period (*p* = 0.002, *p* < 0.001, respectively). All results are summarized in Table 2.

### 2.2. Comparison of Sleep-Related Indices before and after Treatment

**Figure 1:**
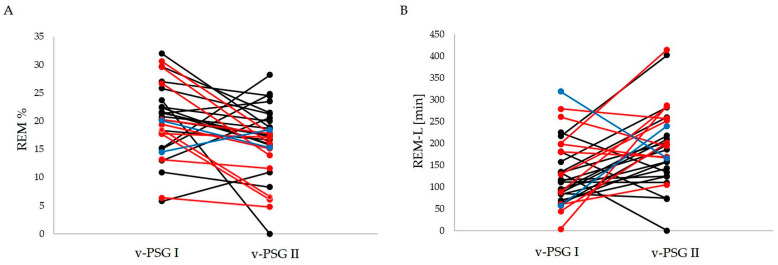
Within-group comparisons of significantly changed parameters before and after treatment: (**A**) REM%; (**B**) REM latency (min). Black marks and lines indicate the patients with a drug dose of 10 mg; red marks and lines indicate the patients with a drug dose of 15 mg; blue marks and lines indicate the patients with a drug dose of 20 mg; REM—rapid eye movement, v-PSG I—video polysomnography before treatment, v-PSG II—video polysomnography after treatment.

Index REM-L was significantly higher after treatment compared to before the treatment period (v-PSG I) (*p* = 0.005), while index REM% was significantly decreased after treatment (v-PSG II) compared to before the treatment period (*p* = 0.009). The remaining evaluated sleep-related parameters (RD, TST, WASO, SE%, SL, PLMS index (PLMI), AI, REM fragmentation, N1%, N2%, and N3%) displayed no significant treatment effects. All results are summarized in Table 3. Moreover, the within-group comparison of significantly changed parameters is presented in Figure 1.

In addition, there was no significant treatment effect in spectral power indices of delta and alpha waves during all sleep periods, as well as during individual sleep stages (N1, N2, N3, NREM, and REM).

## 3. Discussion

This study explored the effects of vortioxetine on the sleep architecture of adolescents with MDD using polysomnography for the first time. The major findings are the following: (1) total scores of both evaluated questionnaires (CDI and AIS) were significantly reduced after VOR treatment; (2) REM latency was significantly higher after treatment (v-PSG II) compared to before the treatment period (v-PSG I); and (3) total REM percentage decreased significantly after treatment compared to v-PSG I. These results indicate the dose-dependent REM-suppressing effect and clinical antidepressant effect of VOR in adolescent patients. Several mechanisms are suggested.

Firstly, we can assume similar effects derived from limited sleep data in adults and the pharmacodynamic profile of VOR. Specifically, in adults, according to a randomized double-blind study of healthy males, it may increase WASO (in a higher dose of 40 mg/day), increase N1 stage duration, and more importantly reduce REM sleep [17,18]. The REM-suppressing effect is attributed to 5-HT_1A_ receptor agonism and 5-HT_7_ antagonism, but is less pronounced compared to SSRIs—probably as the result of 5-HT_3_ antagonism and higher affinity to this receptor [18,30]. The possible explanation of the attenuated effect on REM suppression is that the antagonist interaction of VOR on the 5-HT_3_ receptor increases serotonin levels and induces the cholinergic REM-ON neurons, thus stimulating the REM sleep onset [17,30]. Also, the 5-HT_3_ receptors are ion channels present in the GABAergic subpopulation of interneurons located in the medial prefrontal cortex and hippocampus—the brain structures that are highly connected to mood, behavior, and cognitive functions [31]. Another favorable effect is antagonism on 5-HT_7_, which prevents sleep fragmentation (the increase of arousals during sleep) as observed in SSRIs [30,32]. Overall, it has not been yet fully understood how 5-HT receptors are involved in sleep–wake regulation and how VOR participates through its interactions with these receptors. REM disinhibition in depressed adolescents is associated with a subjective perception of impaired sleep [2]. One of several possible pathomechanisms is persisting hyperarousal activity linked to emotional, mood, and cognitive dysregulation common in patients with insomnia and depression [9]. Taking into account this relationship and previously referred improvement of subjective sleep quality in adults during VOR treatment [27,28], this study revealed similar findings by improvement of the total CDI and AIS scores. In terms of objective polysomnography data, an increased REM latency associated with decreased total REM sleep percentage, but surprisingly without significant changes in REM density, could indicate REM-suppressing effects of VOR. Our results are consistent with another study comparing VOR to paroxetine (an SSRI) and placebo in animal models [30] and healthy humans [17], which found REM-suppressing effects of VOR to be associated with increased synaptic 5-HT concentration [30]. However, it is important to note that REM-L has been already prolonged before VOR treatment, which is in contrast with previous studies reporting shorter REM-L in depressed adolescents [33,34]. Furthermore, we did not find a significant effect of VOR on sleep continuity (i.e., prolonged sleep latency, increased wakefulness after sleep onset, arousal index, PLMI, etc.) in the administered dose range. However, it is important to note that PLMI, at baseline, can point to PLMS syndrome (considering PLMI ≥ 15/h [35]) in depressed adolescents. To sum up, a predominance of REM sleep within sleep cycles is considered a transdiagnostic phenomenon that can be observed across different neuropsychiatric disorders [4]. REM sleep abnormalities could be interpreted as an etiological factor or as a consequence of pathological processes determining depressive disorder as a part of maladaptive stress response [2,3,4]. Thus, the REM-suppressing effect of most serotoninergic ADs indicates a therapeutic response [36], which is consistent with our findings. VOR has the potential to modulate sleep structure in a distinct way compared to SSRIs, most likely due to its multi-target pharmacological profile. With regards to selected sleep biomarkers (such as REM latency, REM sleep percentage, REM fragmentation, and REM density), we suspect different long-term results of VOR treatment, identifying non-responders or even predicting relapse.

### Limitations of the Study

There are several limitations of this study. The study was based on a relatively small sample size of predominantly girls; therefore, further research is needed with respect to sample size and sex. Additionally, the data were collected from a single scheduled night of v-PSG without considering night-to-night variability and individual circadian preferences common for the adolescent age. There is another important aspect of potentially different effects of acute and (sub)chronic treatment with VOR; therefore, it is important to follow up on the stability of related sleep variables.

## 4. Materials and Methods

### 4.1. Ethical Statement

This research was approved by the local Ethics Committee of the Jessenius Faculty of Medicine in Martin, Comenius University, and accomplished in accordance with the ethical principles of the Declaration of Helsinki (1975).

### 4.2. Participants

**Figure 2:**
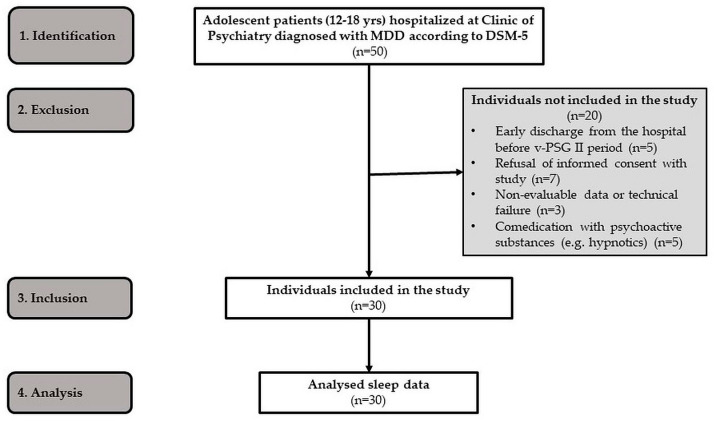
STROBE flow chart of participants.

We screened 50 adolescent patients hospitalized at the Clinic of Psychiatry. The inclusion criteria are as follows: (1) the diagnosis of MDD according to DSM-5 criteria (APA, 2013 [37]); (2) the adolescent age (aged 10 to 19 years according to WHO (World Health Organization, 2023) [38]); (3) depressive episode (moderate to severe) without psychotic symptoms; (4) duration of MDD less than three months; (5) discontinuation of previous antidepressant medication (i.e., SSRI—fluoxetine)—the wash-out period was two weeks; (6) negative pregnancy test for girls; and (7) obtained informed consent from the legal representative of the patients. The exclusion criteria were as follows: (1) comorbid disorders (of other mental disorders, neurologic, metabolic, or sleep disorders); (2) co-medication with psychoactive substances (i.e., benzodiazepines, hypnotics, psychostimulants, antipsychotics, and other psychotropic substances); and (3) co-intervention during the study (i.e., psychotherapy). Therefore, the final study group consisted of 30 MDD adolescents (12–18 years; mean age 15 years ± 1.5 SD; 21 girls), who were, at the time, hospitalized at the Clinic of Psychiatry, Jessenius Faculty of Medicine in Martin and University Hospital Martin, Slovak Republic (Figure 2, Table 4). The written informed consent was obtained from the parents of participants. All participants were diagnosed with major depressive disorder (MDD) and some of them presented suicidal ideas at the time of admission. The MDD was diagnosed according to the criteria from the Diagnostic and Statistical Manual of Mental Disorders, Fifth Edition (DSM-5, 2013 [37]), by two independent specialists.

### 4.3. Clinical Assessment

A series of self-reported scales focusing on core symptoms of depression (such as anxiety, depressive mood, and insomnia) at the time of v-PSG I and v-PSG II recordings were provided by specialists in child and adolescent psychiatry. The self-reported scales included the Children´s Depression Inventory (CDI) and the Athens Insomnia Scale (AIS). 

#### 4.3.1. The Children´s Depression Inventory

The CDI is a self-report scale that has fulfilled the criteria of validity and reliability for measuring symptoms of depression in children and adolescents (from 7 to 17 years) [39]. The scale measures a range of depressive symptoms including the following items: disturbed sleep, hedonic capacity, vegetative functions, self-evaluation, and interpersonal behavior. The CDI includes 27 items rated on a scale of 0 (absence of symptom) to 2 (definite symptom), and the total score ranges from 0 to 54. The recommended cut-off score is 13 for the clinical population and the cut-off score that differentiates between the depressive and general population is 19 [40]. The total CDI score was assessed in participants before and after the antidepressant treatment period. 

#### 4.3.2. The Athens Insomnia Scale

The AIS is a self-reported reliable and validated scale for screening and assessment of the severity of insomnia in adolescents. It contains eight items each rated between 0 and 3 (with 0 corresponding to no problem at all and 3 to a very serious problem). The scale measures difficulties with sleep initiation, awakenings during the night, early morning awakenings, total sleep time, and the overall quality of sleep, while the last three items refer to well-being, functioning capacity, and sleepiness during the day. It has high internal consistency (α = 0.81) and reliability (r = 0.46–0.66 and 0.80, respectively) with an optimal cut-off score of 6 points [41]. The total AIS score was assessed before and after the antidepressant treatment period.

The questionnaires were administered by two independent specialists in child and adolescent psychiatry (Kovacova V., M.D. and Macejova A., M.D.).

### 4.4. Protocol 

**Figure 3:**
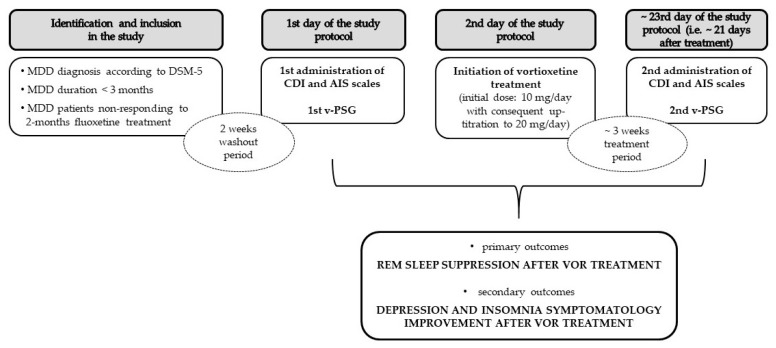
Study flow diagram.MDD—major depressive disorder, DSM-5—Diagnostic and Statistical Manual of, CDI—Children´s Depression Inventory, AIS—Athens Insomnia Scale, v-PSG—video-polysomnography, REM—rapid eye movement sleep.

All participants underwent two attended v-PSG recordings before and after antidepressant therapy according to the given sleep schedule (from 9 p.m. to 5 a.m.) without an adaptation night. All participants discontinued taking previous antidepressant medication (SSRI—fluoxetine) with a two-week drug-free period prior to the first overnight video-polysomnographic evaluation (v-PSG I) according to the long elimination half-life of the active metabolite of fluoxetine (i.e., norfluoxetine), which ranges from 7 to 15 days [42]. Considering that the time to onset of the antidepressant effect of VOR is approximately 2 weeks [43], we scheduled AIS and CDI scoring and a second video-polysomnography (v-PSG II) after ~3 weeks of VOR treatment (Figure 3).

Polysomnography data were acquired by using Sleepware G3 with Somnolyzer software (Philips Respironics, Canada), set and scored by two independent and trained specialists, who were blinded to the participants´ clinical conditions (Mlyncekova Z., M.D. and Hutka P., M.D., Ph.D.), according to the American Academy of Sleep Medicine (AASM) guidelines (manual version 2.4; 2017) [44]. Recordings included standard EEG, electrooculography (EOG), bilateral mentalis and anterior tibialis muscles electromyography (EMG), electrocardiography (ECG), nasal and oral airflow thermistor, thoracoabdominal bands, oxygen saturation, and continual video monitoring. 

The polysomnography data monitored from two nights were used in the analysis of visually scored sleep stages, and then for power spectral analysis. The conventional sleep-related indices derived from visual scoring were the following: total sleep time (TST), wake after sleep onset (WASO), sleep efficiency (SE), sleep latency (SL), REM latency (REM-L), and percentage of time spent in NREM stages (N1, N2, and N3), and REM sleep percentage (REM %). REM density (RD) was calculated as the total number of rapid eye movements per minute of REM sleep. Next, PLMI was automatically scored as the number of limb movements per hour of total sleep. Further, the parameters of sleep fragmentation—REM fragmentation and arousal index (AI)—were calculated as the number of REM sleep awakenings per hour of REM sleep [45] and the number of arousals per hour of sleep [46], respectively. 

Additionally, the sleep montage, used in this study, consisted of a single channel of EEG (Cz referenced to M). For EEG monitoring, high- and low-frequency filter settings were 100 and 0.3 Hz, respectively, and the 60-Hz notch filter was activated. EEG signals were first low-pass filtered with an anti-aliasing filter (70 Hz, 24 dB/octave). Then, the EEG was converted from analog to digital form with a sampling rate of 256 Hz. Digital EEG signals were band-limited to 50 Hz by a digital finite impulse response (FIR) filter before being decimated from 256 to 128 Hz [47,48]. Consequently, digitized EEG signals were analyzed with power spectral analysis. Awake time and artifacts were excluded from these analyses. The EEG delta and alpha activities power densities in μV2 were calculated by automatic software Sleepware G3 Somnolyzer powers (Philips), from the 128-Hz signals for consecutive 4-s epochs in 0.25-Hz frequency bandwidths, using a fast Fourier transform (FFT). Artifact-free 4-s power density values were used to calculate mean power density for 0.25-Hz bandwidths during the all-sleep period, and individual sleep stages (N1, N2, N3, NREM, and REM) across the whole sleep period.

### 4.5. Pharmacotherapy

All patients with a primary diagnosis of MDD were initially treated with the recommended antidepressant treatment at the adolescent age—i.e., the SSRI medication fluoxetine. The switch to “off-label” VOR treatment in MDD adolescents was indicated in patients who had not responded to a 2-month initial treatment with fluoxetine. VOR was administered orally once a day in a dose of 10/15/20 mg per day, with the initial dose of 10 mg/day, and following up-titration to 20 mg/day after 1 week in those patients who required higher dosages, starting the first day after v-PSG I night. Considering that the time to onset of the antidepressant effect of VOR is approximately 2 weeks [43], we scheduled the second AIS and CDI scorings and v-PSG II at least 2 weeks after treatment initiation, exactly 21 (18, 26) days.

### 4.6. Statistical Analysis

The data were explored and analyzed in jamovi version 1.6.9 (Sydney, Australia). Data distributions (Gaussian/non-Gaussian) were assessed by the Shapiro–Wilk normality test. After normality testing, a Student´s *t*-test for related Gaussian distributed variables or a Wilcoxon-rank test for non-Gaussian data with size effect estimations by Cohen´s d was applied to comparison periods before and after treatment. Next, the necessary sample size was calculated to be 28, where the confidence level was 95%, the standard deviation was 0.5, and the margin of error was 5%. Moreover, for index REM-L, a sample size of 24 subjects, 12 in each group, is sufficient to detect a clinically important difference of 45 min between groups, assuming a standard deviation of 30 min using a two-tailed *t*-test of difference between means with 95% power and a 5% level of significance. Considering a dropout rate of 10%, the sample size required is 26 (13 per group). For index REM%, a sample size of 22 subjects, 11 in each group, is sufficient to detect a clinically important difference of 4% between groups, assuming a standard deviation of 2.5% using a two-tailed *t*-test of difference between means with 95% power and a 5% level of significance. Considering a dropout rate of 10%, the sample size required is 24 (12 per group). Data are expressed as mean ± standard deviation (SD). A value of *p* < 0.05 (two-tailed) was considered statistically significant.

## 5. Conclusions

This study in adolescent patients with MDD revealed significant REM-suppressing effects (an increased REM latency and a reduced REM sleep percentage) after VOR treatment, presumably dose-related. We suggest that our findings could contribute to the understanding of the interaction between VOR and sleep, which is important for rational pharmacotherapy in adolescent patients suffering from MDD.

## Figures and Tables

**Table 1:** Vortioxetine and its effects on sleep.

Study	*n* (Number of Subjects)	Study Design	Treatment Duration Dosing Regimen	Results
Liguori et al., 2019 [27]	15 adults with MDD and insomnia	A retrospective analysis of questionnaires (PSQI, ESS, BDI)	6 months VOR at 10 mg	Improvements in subjective sleep complaints and reduction of depressive symptoms
Cao et al., 2019 [28]	92 adults with MDD and 54 healthy controls	A post-hoc analysis of the clinical trial of sleep questionnaires (PSQI, ESS, ISI)	8 weeks VOR at 10–20 mg	Improvements in sleep (predictive of AD response) and linearly correlated to depressive symptoms
Leiser et al., 2015 [30]	Animals (rats)	Bipolar sleep EEG	1, 3, 7, 10 days VOR at 0.6 mg/kg (s.c. injection, drug-infused chow, or water)	↓ REM sleep % (only acute effect)
Wilson et al., 2015 [17]	19 healthy men	A randomized, double-blind, placebo-controlled (compared to paroxetine and placebo) PSG study	2 days VOR at 20–40 mg paroxetine at 20 mg	~↑ REM-L, ~↓ REM sleep % ↑ N1 stage % ~↑ WASO (at 40 mg dose)

AD—antidepressants, EEG—electroencephalography, PSG—polysomnographic, VOR—vortioxetine, PSQI—the Pittsburgh Sleep Quality Index, ISI—the Insomnia Severity Index, ESS—the Epworth Sleepiness Scale, BDI—the Beck Depression Inventory, REM—rapid eye movement sleep, REM-L—rapid eye movement sleep latency, MDD—major depressive disorder, WASO—wake after sleep onset. ↑—increased sleep variable, ↓—decreased sleep variable, ~ dose-dependent effect on the evaluated sleep variable.

**Table 2:** The questionnaire indices before and after treatment.

	Before Treatment	After Treatment	*p*-Value	Cohen’s d
AIS	7.53 ± 5.73	4.70 ± 3.91	0.002	0.6589
CDI_A	5.10 ± 2.80	3.00 ± 2.74	<0.001	0.8866
CDI_B	1.93 ± 1.41	1.70 ± 1.11	0.007	0.6052
CDI_C	3.60 ± 2.27	2.70 ± 1.91	0.025	0.4412
CDI_D	6.10 ± 3.61	4.47 ± 3.12	0.013	0.5508
CDI_E	4.17 ± 2.61	2.83 ± 2.55	0.002	0.6536
CDI_TS	20.9 ± 11.3	14.1 ± 10.0	<0.001	0.8061

AIS—the Athens Insomnia Scale, CDI—the Children’s Depression Inventory, CDI_TS—the Children´s Depression Inventory total score, v-PSG I—before treatment period video-polysomnography, v-PSG II—after treatment period video-polysomnography. All data are summarized as mean ± SD. The value of *p* < 0.05 is considered statistically significant.

**Table 3:** Mean differences in conventional sleep-related indices, sleep fragmentation indices, and EEG spectral power for delta and alpha wave during all sleep periods and individual sleep stages (N1, N2, N3, NREM, and REM) before and after treatment.

	v-PSG I	v-PSG II	*p*-Value	Cohen’s d
Conventional sleep-related indices				
TST (min)	404.0 ± 49.9	391.0 ± 70.9	0.363	0.1689
WASO	51.4 ± 28.9	70.1 ± 57.4	0.152	−0.3190
SE%	79.9 ± 9.34	76.1 ± 13.4	0.152	0.2884
SL	50.8 ± 39.1	52.2 ± 35.9	0.887	−0.0322
REM-L	132.0 ± 75.3	193.0 ± 89.8	0.005	−0.5569
REM%	20.10 ± 6.59	16.20 ± 6.33	0.009	0.5086
RD	3.12 ± 2.80	2.74 ± 2.29	0.931	−0.0207
N1%	4.67 ± 2.22	6.00 ± 4.41	0.234	−0.2951
N2%	47.10 ± 8.80	48.10 ± 8.76	0.461	−0.1364
N3%	28.20 ± 7.99	29.60 ± 10.90	0.551	−0.1478
PLMI	17.70 ± 21.30	32.00 ± 37.10	0.119	−0.3320
Sleep fragmentation indices				
REM fragmentation	11.70 ± 7.07	12.50 ± 7.12	0.558	−0.0997
AI	14.40 ± 7.05	17.50 ± 10.00	0.318	−0.2129
Spectral power indices				
Delta power (μV^2^)	7.38 ± 0.19	7.40 ± 0.18	0.632	−0.0933
Delta power N1 (μV^2^)	5.35 ± 0.45	5.38 ± 0.49	0.824	−0.0431
Delta power N2 (μV^2^)	6.76 ± 0.25	6.77 ± 0.21	0.916	0.0205
Delta power N3 (μV^2^)	7.21 ± 0.20	7.24 ± 0.20	0.396	−0.1660
Delta power NREM (μV^2^)	7.36 ± 0.18	7.38 ± 0.18	0.512	−0.1280
Delta power REM (μV^2^)	5.88 ± 0.45	5.76 ± 0.43	0.382	0.1745
Alpha power (μV^2^)	6.70 ± 0.04	6.74 ± 0.22	0.257	−0.2232
Alpha power N1 (μV^2^)	5.03 ± 0.49	5.06 ± 0.56	0.540	−0.1217
Alpha power N2 (μV^2^)	6.52 ± 0.24	6.54 ± 0.28	0.768	−0.0575
Alpha power N3 (μV^2^)	5.94 ± 0.48	6.03 ± 0.35	0.171	−0.2710
Alpha power NREM (μV^2^)	6.67 ± 0.24	6.71 ± 0.22	0.253	−0.2247
Alpha power REM (μV^2^)	5.17 ± 0.62	5.06 ± 0.76	0.374	0.1776

v-PSG I—before treatment period video-polysomnography, v-PSG II—after treatment period video-polysomnography, TST—total sleep time, WASO—wake after sleep onset, SE%—sleep efficiency, SL—sleep latency, PLMI—number of periodic limb movements in total sleep per hour, AI—arousal index, REM—rapid eye movement sleep, REM-L—REM latency, REM%—REM sleep percentage, RD—REM density, N1%—N1 stage percentage, N2%—N2 stage percentage, N3%—N3 stage percentage. All data are summarized as mean ± SD. Note:The value of *p* < 0.05 is considered statistically significant.

**Table 4:** Baseline characteristics of MDD adolescents involved in the study.

Characteristics of MDD Adolescents (*n* = 30)	
Age (mean ± SD)	15.0 ± 1.5 years
Female/male	21/9
Duration of MDD	<3 months
Drugs used prior to VOR	Fluoxetine
Severity of MDD	Moderate or severe
Comorbidities	None
Co-interventions	None
VOR—10 mg/day	18 adolescents
VOR—15 mg/day	10 adolescents
VOR—20 mg/day	2 adolescents

MDD—major depressive disorder, VOR—vortioxetine.

## Data Availability

Complete data are available upon reasonable request from the corresponding author.
